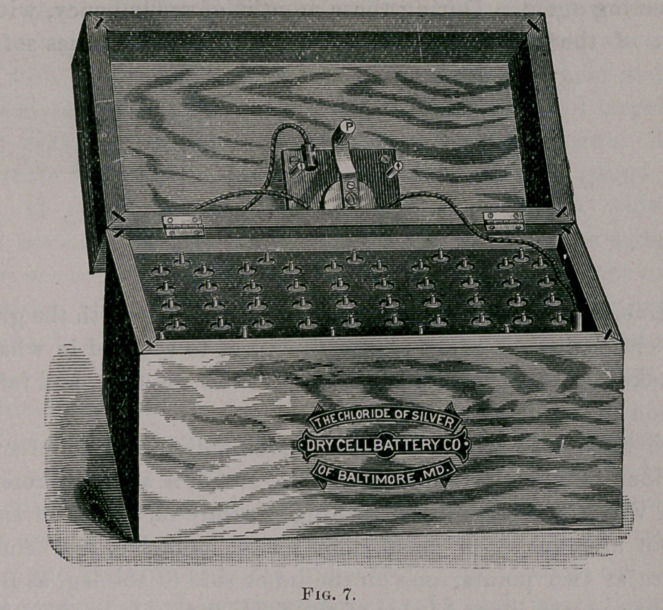# Electricity in the Diagnosis of Diseases of the Nervous System

**Published:** 1892-10

**Authors:** Frederick Peterson

**Affiliations:** Chief of Clinic, Nervous Department, College of Physicians and Surgeons; Attending Physician to the New York Hospital for Nervous Diseases; 201 West Fifty-fourth Street, New York


### ELECTRICITY IN THE DIAGNOSIS OF DISEASES OF THE NERVOUS SYSTEM.

By FREDERICK PETERSON, M. D.,

Chief of Clinic, Nervous Department, College of Physicians and Surgeons; Attending Physician to the New York Hospital for Nervous Diseases.

The value of electricity to the neurologist, as an agent in diagnosis, is, I believe, little known or understood outside of the ranks of specialists. It would seem, therefore, that a clear and explicit statement of the points and facts of electro-diagnosis would prove valuable to the general medical practitioner. We use electricity to obtain a positive symptom in tetany, exophthalmic goitre, and hysteria ; to test all of the five senses — taste, smell, sight, hearing, and feeling ; to test the ciliospinal reaction ; to determine the situation of a lesion in a facial neuralgia ; to localize centers for brain and spine surgery ; and finally, and most commonly, to distinguish paralyses due to lesions in the spinomuscular segment of the motor tract.

*Tetany.*—A positive symptom of tetany is the increased reaction of the muscles to the galvanic current. Their electrical irritability is so astonishingly great, that a current from two cells, or sometimes even from one cell, is sufficient to cause contractions in the muscles. This is one of the cardinal symptoms, therefore, of tetany.

*Basedow's Disease or Exophthalmic Goitre.*—In this disease the resistance to the galvanic current is greatly diminished, probably owing to the hyperidrosis and superficial capillary dilatation generally manifested. One sponge electrode being placed on the chest and another on the back, the current is allowed to pass until the galvanometer registers, say, five or ten milliamperes. Then a wire-coil rheostat is substituted for the human body, and coil after coil is introduced into the circuit until the number of milliamperes corresponds with that previously obtained. Then one reads off the number of ohms marked upon the rheostat, which gives us, naturally, the ohms resistance of the human body. The normal average resistance of the human body may be given as from 2,000 to 4,000 ohms. In exophthalmic goitre it is reduced, in a majority of cases, to between 1,000 and 2,000 ohms, or about half. This reduction of resistance is a positive symptom when present ; but it has no negative value if not present.

*Hysteria.*—Vigouroux has recently pointed out that the resistance in hysteria is increased above the average normal resistance. In hysterical hemianesthesia the resistance on the anesthetic side is greater than on the opposite side.

TESTING THE FIVE SENSES.

*Taste.*—This sense may be examined for the galvanic taste by applying the small wire ends of the rheophores closely approximated to different parts of the tongue. A current from one or two cells suffices. A sharp metallic taste, known as the "galvanic taste," is produced. A Steiner electrode, such as is used to stimulate the cortex of the brain, may be also used for this purpose. The "galvanic taste" is absent on the front of the tongue in injury to the chorda tympani, and on the back of the tongue in lesions of the glossopharyngeal nerve.[1]

*Smell.*—The sense of smell may be tested by wrapping the end of one rheophore well in absorbent cotton, saturated with water, and inserting it into the nostril to be examined. The other pole may be in the hand. One to five cells are used. A peculiar phosphoric odor is perceived if the olfactory nerve is intact.

*Sight.*—Whenever electrodes are applied to the head, especially in the neighborhood of the eyes, flashes of light are perceived when the current is made or broken. These are not observed in total blindness. A mild galvanic current is employed. It is a delicate test of the condition of the retina, sometimes showing faint reactions when light has no apparent stimulus.

*Hearing.*—If one sponge electrode be placed over the ear and one on the nape of the neck, a sound will be heard, usually of a ringing character, a clang, when the current is made. The loudest sound is made on closure of the cathode. There is also a weak sound with the opening of the anode. There is a galvanic hyperesthesia in many cases of central disease. This is especially true in insanity with auditory hallucinations. In certain cases there is a change of the formula—a sound heard with the cathodal opening and anodal closure, or even a reversion of the formula.

*Cutaneous Anesthesia.*—With the wire brush (Fig. 1) and a mild faradic current we may demarcate areas of anesthesia of tactile and pain senses.

FIG. 1.

CILIO-SPINAL PUPILLARY REACTION.

By stimulating the skin of the neck with the faradic brush, we, under normal conditions, dilate the pupil on the same side by the reflex through the cilio-spinal center (in fourth cervical to second dorsal segments of spinal cord). In destruction of this center, for instance, in Klumpke's paralysis, or in lesion of any part of the reflex path, this reaction is lost.

1. See author's paper, Disturbance of the Sense of Taste after Amputation of the Tongue, *N. Y. Medical Record*, August 30, 1890.

### TO DETERMINE WHETHER SEVERE NEURALGIA IN SUPERFICIAL NERVES IS PERIPHERAL OR CENTRAL.

*Cocaine Cataphoresis.*— A ten to twenty per cent. solution of cocaine, on a cataphoric anode, applied with a continuous current to a painful nerve, such as a branch of the trigeminal, will, in a few minutes, anesthetize the nerve and relieve the pain, if the lesion is beneath or peripheral to the electrode. Thus, an intense infra-orbital neuralgia, if not relieved by cocaine cataphoresis, is probably of central origin, or, perhaps, hysterical in nature, and neurectomy is contra-indicated.[1]

### IN LOCALIZING CORTICAL CENTERS IN BRAIN SURGERY.

We have here a very important use for the faradic current, with a Steiner electrode; for, in locating the parts we want to excise, it is essential that we orient ourselves as to the parts we have trephined over. Fortunately, we are able, in this way, to recognize our surroundings before opening the dura mater, for I have stimulated the cortical centers through the dura, in experiments upon a monkey, at the Laboratory of the College of Physicians and Surgeons, and also upon man, in brain operations undertaken at Charity Hospital. It would be of still greater advantage were we able to stimulate centers through the skull, underneath the scalp. I experimented upon some animals, at the Edison Laboratory, this Summer, in conjunction with Mr. Kennelly, with a variety of currents, to determine this point. We were unable to excite cortical centers through the skull bones after removal of the scalp, but this is not owing to the bone being a bad conductor, as it is generally supposed to be, but because, as we were able definitely to decide, the skull bones are such good conductors that currents are short-circuited and do not reach the brain substance.

### IN LOCALIZING SPINAL CENTERS IN SPINAL SURGERY.

The Steiner electrode, with mild faradic currents, may be employed with advantage here also, in stimulating and thus local-

1. See the following articles by the author: Electric Cataphoresis as a Therapeutic Measure, *N. Y. Medical Journal*, April 2, 1889; A System of Exact Dosage in the Cataphoretic Use of Drugs, *N. Y. Medical Journal*, November 15, 1890; A Further Study of Anodal Diffusion as a Therapeutic Agent, *N. Y. Medical Record*, January 31, 1891; The Introduction of Drugs into the Body by Electricity, *Philadelphia Medical Times and Register*, March 21, 1891.

izing and recognizing spinal nerve-roots and segments, during operations upon the vertebral canal.

IN DISTINGUISHING PERIPHERAL PARALYSES.

Probably, however, the most useful application of electricity, from a diagnostic point of view, is to differentiate paralyses due to a lesion of the spino-muscular portion of the motor tract from paralyses due to lesions in the central nervous system, affecting the cortico-spinal portion of the motor tract.

For this purpose, both the galvanic and faradic currents are generally employed, but practically it is usually sufficient to use the faradic current alone.

*Normal Reactions in Nerves and Muscles.*—When any current is suddenly applied to the motor point of a nerve or muscle, contraction takes place in the muscle. This is true under normal conditions, and it is also true in any paralysis due to lesion in any part of the spinal cord or brain affecting cortico-spinal motor fibers.

To obtain these reactions, a flat sponge electrode (Fig. 2) is placed

FIG. 2.

upon any indifferent part of the body, say the back, or chest, or in the hand. Another electrode, with an interrupting handle (Figs. 3

FIG. 3.

and 4) is then applied to a motor point. In using the faradic current we need make no distinction between the poles, whether they are negative or positive. The results are precisely the same. The fact is, that with the primary current the cathodal contractions are a trifle more powerful than the anodal ; while with the secondary there is absolutely no difference, because the secondary current is an alternating current, each pole being first positive, then negative, with the rapid to-and-fro movement of the electric waves.

FIG. 4.

But when the galvanic current is used, there is a difference in the amplitude of contraction produced by the two poles. Hence, it is important, indeed absolutely necessary, in employing this current, always to distinguish the positive pole or anode from the negative pole or cathode. In normal muscles, the cathode produces a stronger contraction than the anode. Having made our interrupting handle on the motor point the cathode, we increase the number of galvanic cells until our closures of the circuit produce a slight contraction. This is called the cathodal closure contraction (CCC). Now, if the current-reverser be moved, so that the interrupting handle is made the anode, it will be found that the current is not yet strong enough to produce an anodal closure contraction (AnCC), but the muscle may contract just as you open the circuit, making an anodal opening contraction (AnOC). A stronger current gives us the anodal closure contraction, and with a very strong current we may obtain with the cathode also an opening contraction (COC). Thus, beginning with a weak current, and gradually increasing it, we note first a CCC, then in addition AnOC, later AnCC also, and finally, with very strong currents, we have contractions with either pole both upon opening and closing the circuit. It is not necessary, for our purposes, to pay attention to the opening contractions. The "normal formula" for us, practically, is that the cathodal closure contraction is stronger than the anodal closure contraction (CCC>AnCC).

*Reaction of Degeneration.*—Whenever there is degeneration of a nerve and the muscles supplied by it, their reactions to electricity are changed. If the degeneration is very slight, there is still reaction, but usually less than will be found in the normal nerves and muscles of the patient examined, so that we speak of a diminished electrical reaction. This is called a quantitative or modal change, and is not of the greatest value for diagnosis. But where degeneration is marked and typical, there is no reaction at all, with either the faradic or galvanic current, when the interrupting handle is placed over the *motor nerve.* Now, when the electrode is applied to the motor point of each suspected *muscle*, it is found not to contract at all to faradism, no matter how strong the current employed. For diagnosis this would be sufficient. Finding that the muscle does not respond to faradism, we know that the reaction of degeneration is present. Still, if we desire to try also the effects of the galvanic current, we find that the muscle does contract with the galvanic current in spite of not doing so with the faradic, only there is a reversion of the normal formula, for now the anodal closure contraction is equal to or markedly stronger than the cathodal closure contraction (AnCC > CCC).

In a nutshell, then, we may describe the complete reaction of degeneration as follows :

| NORMAL MUSCLE. | DEGENERATED MUSCLE. |
|---|---|
| Contracts with faradism. | Does not contract with faradism. |
| Contracts with galvanism. | Contracts with galvanism. |
| (CCC > AnCC.) | (AnCC > CCC.) |

But, now, what do we mean by, and where do we find, degeneration ? In order to clearly understand this we must describe, briefly, the motor tract. This tract extends from the motor area of the cortex to the muscles, but it has two segments. The one, called the cerebro-spinal segment, consists of the motor cells in the cortex and the long fibers going down from it through the brain and spinal cord into the cells of the anterior horns of the cord. The other segment, the spino-muscular, consists of the large nutritive ganglion cells in the anterior horns of the cord and the fibers passing out therefrom to end in the muscles. This spino-muscular segment governs the nutrition of the muscles, while the upper portion, or cortico-spinal, conveys chiefly voluntary motor impulses. So, if there is a lesion in the latter, the electrical reactions are unchanged. It is only in lesions of the spino-muscular segment that we have nutritive changes, or degeneration, in the nerves and muscles, and resulting therefrom the electrical reaction of degeneration. The value of electricity in the diagnoses of certain paralyses, therefore, lies in its ability to distinguish for us these two kinds, to tell us whether the lesion is in the cortico-spinal or spino-muscular segment of the motor tract.

Suppose we are called to a case of paralysis. We take with us a small faradic battery (the small one made by J. C. Vetter &

Co. (Fig. 6) is one of the best), or such a one as I have recently had Stammers, of 1424 Broadway, make for me (the smallest battery

FIG. 6.

known, I believe, for the purpose, Fig. 5, $5\frac{3}{4} \times 2\frac{1}{2} \times 2\frac{1}{2}$ inches ), and

FIG. 5.

having tested the reaction of the muscles in the paralyzed members, we consult the following table :

| FARADIC REACTION PRESENT. | NO FARADIC REACTION. |
|---|---|
| Hemiplegia, or | Poliomyelitis. |
| Monoplegia from any cerebral cause, or | Amyotrophic lateral sclerosis. |
| Lateral sclerosis. | Progressive muscular atrophy. |
| | Multiple neuritis. |
| | Lead palsy. |
| | Arsenical neuritis. |
| | Alcoholic neuritis. |
| | Traumatism to nerves. |
| (The lesion is in the cortico-spinal segment of the motor tract.) | (The lesion is in the spino-muscular segment of the motor tract.) |

1 *N. Y. Medical Journal*, August 20, 1892.

*Exceptions.*— Reactions to faradism present in joint atrophies and in milder forms and earliest stages of some other peripheral palsies (such as facial and musculo-spiral at times). Also present in pseudo-hypertrophic paralysis as long as there are sufficient fibers left in the muscle to contract.

FIG. 7.

In making these tests, always compare the symmetrical halves of the body, and take good note of the exceptions mentioned in the table. The best portable galvanic battery is the chloride of silver dry cell, Fig. 7.

201 WEST FIFTY-FOURTH STREET, NEW YORK.

## Figures and Tables

**Fig. 1. f1:**
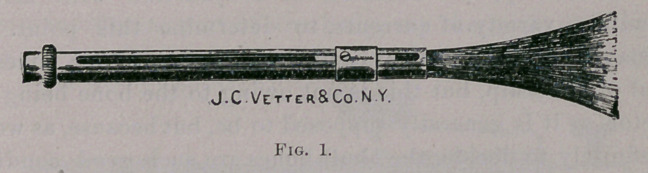


**Fig. 2. f2:**
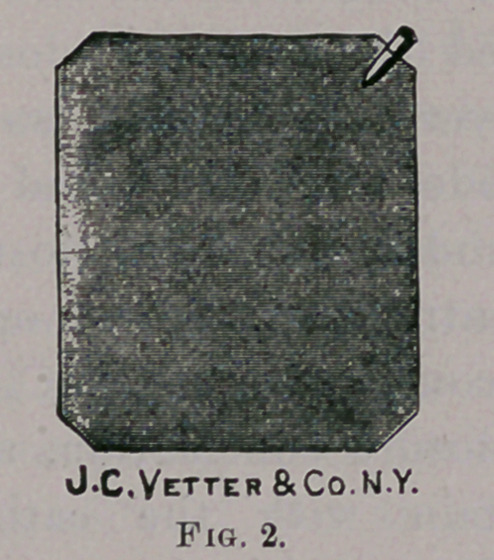


**Fig. 3. f3:**
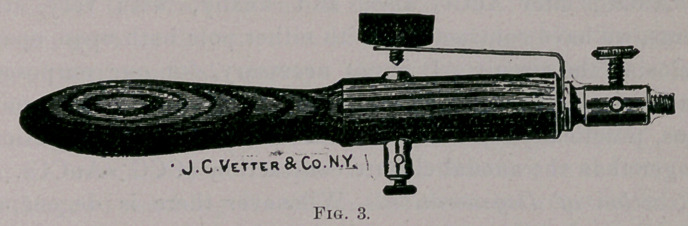


**Fig. 4. f4:**
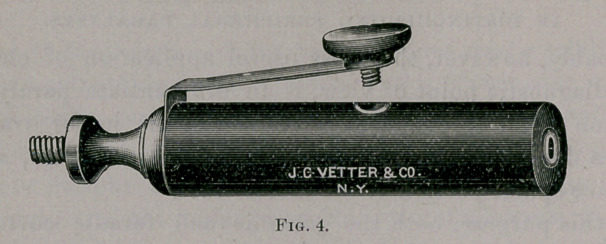


**Fig. 6. f5:**
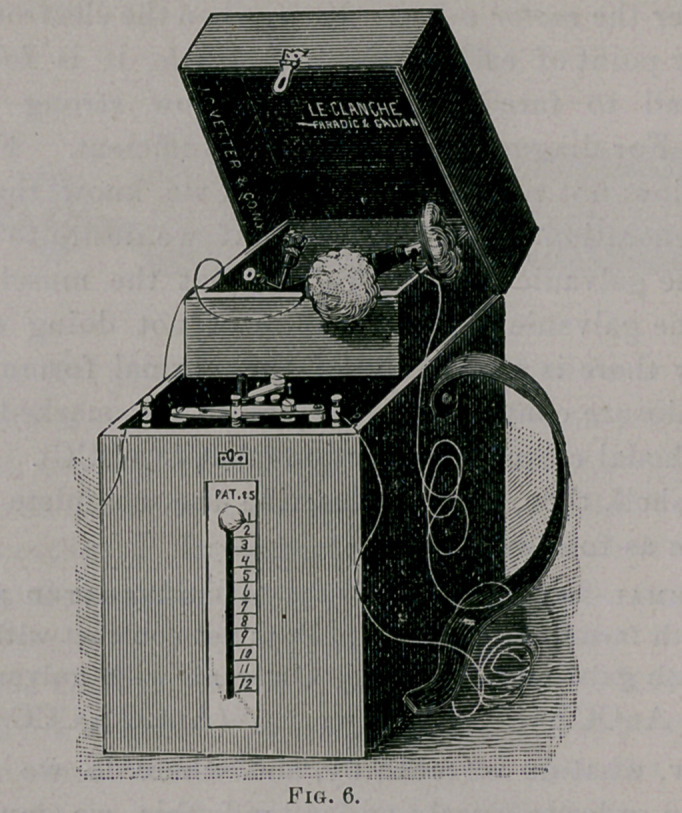


**Fig. 5. f6:**
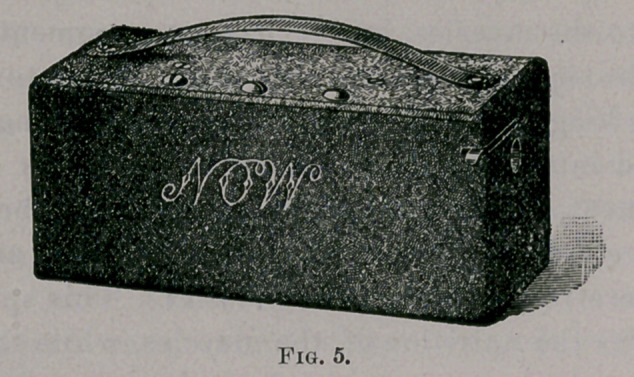


**Fig. 7. f7:**